# Design of Inorganic Polymer Mortar from Ferricalsialic and Calsialic Slags for Indoor Humidity Control

**DOI:** 10.3390/ma9060410

**Published:** 2016-05-24

**Authors:** Elie Kamseu, Isabella Lancellotti, Vincenzo M. Sglavo, Luca Modolo, Cristina Leonelli

**Affiliations:** 1 Department of Engineering “Enzo Ferrari”, University of Modena and Reggio Emilia, Via P. Vivarelli 10, Modena 41125, Italy; kamseuelie2001@yahoo.fr (E.K.); isabella.lancellotti@unimore.it (I.L.); 2 Local Materials Promotion Authority, Nkolbikok Yaoundé 2396, Cameroon; 3 Department of Industrial Engineering, University of Trento, Via Sommarive, 9, Trento 38123, Italy; vincenzo.sglavo@unitn.it; 4 Acciaierie Bertoli Safau S.p.A., Via Buttrio 28–fraz. Cargnacco, Pozzuolo del Friuli (UD) 33050, Italy; L.Modolo@absacciai.it

**Keywords:** steel slag, mix-design, alkali-activated slag (AAS) cements, pore size distribution, microstructure, moisture control capacity

## Abstract

Amorphous silica and alumina of metakaolin are used to adjust the bulk composition of black (BSS) and white (WSS) steel slag to prepare alkali-activated (AAS) mortars consolidated at room temperature. The mix-design also includes also the addition of semi-crystalline matrix of river sand to the metakaolin/steel powders. The results showed that high strength of the steel slag/metakaolin mortars can be achieved with the geopolymerization process which was particularly affected by the metallic iron present into the steel slag. The corrosion of the Fe particles was found to be responsible for porosity in the range between 0.1 and 10 µm. This class of porosity dominated (~31 vol %) the pore network of B compared to W samples (~16 vol %). However, W series remained with the higher cumulative pore volume (0.18 mL/g) compared to B series, with 0.12 mL/g. The maximum flexural strength was 6.89 and 8.51 MPa for the W and B series, respectively. The fracture surface ESEM observations of AAS showed large grains covered with the matrix assuming the good adhesion bonds between the gel-like geopolymer structure mixed with alkali activated steel slag and the residual unreacted portion. The correlation between the metallic iron/Fe oxides content, the pore network development, the strength and microstructure suggested the steel slag's significant action into the strengthening mechanism of consolidated products. These products also showed an interesting adsorption/desorption behavior that suggested their use as coating material to maintain the stability of the indoor relative humidity.

## 1. Introduction

Due to their industrial scale of production, fly ash and slag are now presented as low life cycle CO_2_ emissions, environmentally-friendly and sustainable solid precursors for alkali-activated cement, mortar and concrete [1,2]. A wide variety of such industrial wastes is described in the literature with very variable composition [3,4] thus making their alkali-activation a complex issue. Apart from coal fly ash, blast furnace slag, calcined kaolins, and few others for which it can be said confidently that are suitable for EN 197 cements and can also be properly and successfully employed in the production of geopolymers or hybrid alkaline cements, few recent works have been aimed at valorization of poorly reactive/not-recycled waste materials [5,6]. In the present study two different steel slags were used with metakaolin and sand to generate an aluminosilicatic matrix via alkali activation. This aluminosilicatic matrix was designed to attain a good chemical stability taking into account the existing models on mixed C–S–H (CaO–SiO_2_–H_2_O) structure. In fact, the chemical and mechanical stability of alkali-activated materials is based on some equilibrium between the geopolymer gel phase and the reacted aggregate that constitutes the alkali activated matrix. The stability of silicates-based binders, including ordinary Portland cement, has been found to be improved by the presence of Al ions during the polycondensation reactions [1,7]. Aluminum cations incorporated in C–S–H gel were found to improve the resistance to carbonation. It was observed that all aluminum present in the silicate tetrahedra chains were five-fold coordinated species in interlayer and six-fold coordinated at the surface and theywere incorporated into the amorphous silica phase forming fully condensed tetrahedral Al(-SiO)_3_ sites [1,7]. Myers *et al.* [1] identified in granulated blast furnace slag three distinct tetrahedral Al sites: Q^3^(1Al), Q^4^(3Al) and Q^4^(4Al) which are indicative on the cross-linking degree in the calcium (alkali) aluminosilicate hydrate (C–(N–)A–S–H) gels and the presence of additional highly polymerized aluminosilicate products. For appropriate Si/Al ratio, the amount of non-bridging oxygen (NBO) is a function of SiO_2_/Na_2_O molar ratio. Low value of SiO_2_/Na_2_O is consistent with the increasing number of NBO sites where SiQ^2^ and SiQ^1^ structural units were preferentially formed from silicate chains, dimers and monomers. High SiO_2_/Na_2_O molar ratio generates a decreasing number of NBO sites with structures consisting principally of SiQ^3^ and SiQ^4^ structural units which form silicate 3D frameworks and sheets [7]. When CaO is present, C–S–H is the principal hydration product and primary binding phase; it can be described as a single chain structure that faces polymerization decreasing with the increase of Ca content [8].

While pure geopolymer (N–A–S–H or K–A–S–H) and ordinary Portland cement (C–S–H) phases are generally formed from pure metakaolin and kaolin-calcite respectively, the industrial and natural wastes (fly ash, slag, incinerator bottom ash, volcanic ash, rice husk ash, *etc.*) are complex mixes of geopolymers and OPC forming elements. The high temperature of process, the level of vitrification and melting, the cooling rate and the initial bulk composition will result in the formation of amorphous or partially crystalline phases, as mullite, anorthite, plagioclase, pyroxene *etc.* [9,10,11]. The better crystallized phases will not dissolve even in high alkaline media, thus hindering the polycondensation. Therefore, the appropriate mode to design the alkali-activated materials should not be generalized to a simple activation of finely grounded powder with the alkaline solution, but a more complex mix-design approach is necessary.

Slag from ferrous metallurgy are generally calcium- and iron-rich (40 wt %–52 wt % CaO; 70 wt %–80 wt % FeO; 20 wt %–30 wt % Fe_2_O_3_ [9]) with limited Si and Al content (10 wt %–19 wt % SiO**_2_**; 1 wt %–3 wt % Al_2_O_3_ [9]). The main minerals consist of C_3_S, C_2_S, C_4_AF, C_12_A_7_, RO_ss_ (R = Ca, Fe, Mn, Mg solid solution) and free CaO [9,10,11] embedded in a variable amount of amorphous phase depending on the slag cooling practice. Steel slag has a large amount of non-active compounds such as RO and Fe_3_O_4_. In the high alkaline context, like that of inorganic polymer cement, the non-active components as well as the others crystalline phases of the slag are dissolved in an incongruent manner essentially at their surfaces. With their fine particle size, they seem attractive for the mechanical reinforcement of the matrices through the filler effect and densification. Additionally, they can ideally act as nucleation sites to optimize the reaction of geopolymerization.

Starting from the considerations listed above, the approach used for this research was to consider the slag powder as binder precursor, finer and more amorphous particles, as well as fine aggregates, non-reactive particles. We also operated to obtain a geopolymeric paste with Si/Al close to 2.0–2.5 and Na/Al equal to 1, with the addition of a minimum amount of metakaolin. The larger unreacted slag phases remained embedded in such a matrix as aggregate in a mortar.

The two different steel slags from Electric Arc Furnace (EAF) steelmaking industry (Italy), used in this study, were prepared in an industrial process of production of manufactured aggregates. The presence of slag’s reactive phases, eventually metals, in alkaline environment were supposed to produced geopolymeric cements with a relevant porosity suitable for application as passive phase change humidity control material, which can moderate the indoor moisture. In fact our formulations were expected to be close to vesuvianite, sepiolite and zeolite which are all used as hygroscopic base materials in the manufacturing of moisture control materials for indoor wall coatings. Actually, it is generally observed that materials produced with cold setting processes as cement based mortars, concretes, *etc.* is capable to collect up to 8 wt %–10 wt % of humidity after complete curing and exposure to ambient environment. So for, aside the determination of the adsorption/desorption capacity and the permeable voids assessed with water absorption for these steel slag based inorganic polymer mortars, we also evaluated the morphology and the microstructure using several techniques, in particular X-ray diffraction (XRD), Fourier Infrared spectroscopy (FIT-IR), Mercury Intrusion Porosimeter (MIP), three-point flexural strength and Environmental Scanning Electronic Microscope (ESEM).

## 2. Materials and Methods

### 2.1. Characterization of Ferricalsialic and Calsialic Slags

Two steel slags from EAF steelmaking process were provided by Acciaierie Bertoli Safau in Italy and identified as ABS black (BSS) and ABS white (WSS). The first slag is obtained by the primary process in production of steel (melting of scrap in EAF furnace), while the second is obtained by the secondary metallurgical process (final steel alloy in ladle furnace). Additionally, BSS can be classified as an oxidation slag, while WSS is a reduction slag. Both slags, also indicated as SS, coming out from the furnaces, after cooling process, constitute the raw materials for production of aggregates. The cooling of slags is realized in a two-step process: Firstly the hot material is placed underneath water sprinklers of in order to rapidly reduce its temperature, and second the material is stocked in a pile to conclude the cooling process by air. Especially for black slag, the cooling process is very important and necessary to reduce the amount of free lime and to determine a high mechanical resistance for material. The black slag is generally considered as not suitable for the use in production of cement, while it is considered a good material to produce aggregates for concrete, bituminous conglomerate and civil constructions. Due to its high content of free lime and poor mechanical resistance, the white slag is instead suitable for the production of cement and may substitute the lime itself in stabilization of soils. The steel slags used in this study were provided as manufactured aggregates, obtained by processing the raw materials with the following operations: ageing storage, removing of scrap particles by magnetic separation, reduction of dimension by crushing, calibration of different products by screening.

The chemical compositions of the slag were determined by X-ray fluorescence (ARL™ QUANT'X EDXRF, Thermo Fisher Scientific, Waltham, MA, USA) are presented in Table 1. Both slags had negligible content of C. The S content is 0.3% for BSS and 1.6% for WSS. The sulphur content is reported as SO_3_ according to XRF analysis. The chemical dissolution of white ABS slag performed using concentrated NaOH (8M) (extended description of the test method is reported in Ref. [12]) allowed to determine 35.89 wt % of reactive species, while the remaining 64.11 wt % could be accounted as of aggregates. For the black slag, 72.96 wt %was reactive species while only 27.04 wt % was identified as aggregates.

**Figure 1:**
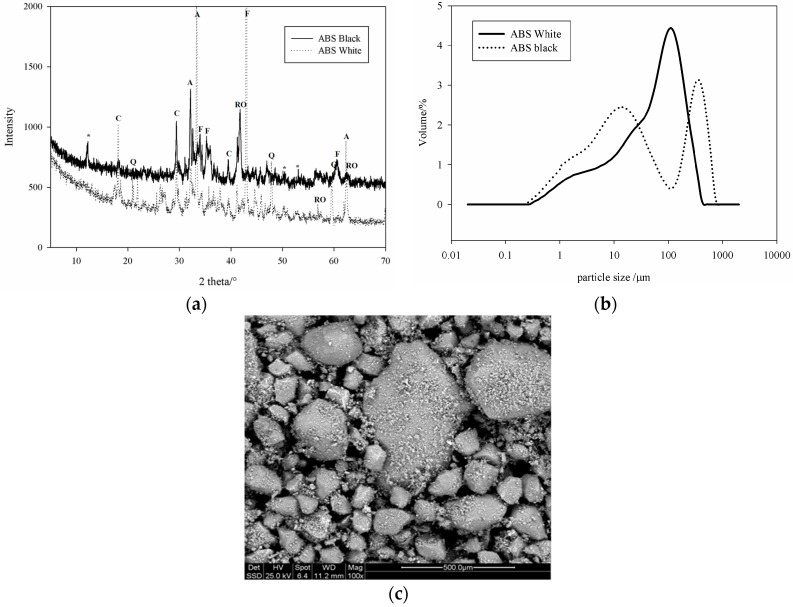
Characterization of the starting two steel slags used for this study: (**a**) XRD patterns (C = Ca(OH)_2_; CF = *x*CaO*_y_*Fe_2_O_3_; CS = C_2_S + C_3_S; Q = quartz; RO = pyroxenes solid-solution); (**b**) grain size distribution; (**c**) powder morphology with ESEM.

The mineralogical phases as determined by XRD analysis, using X-ray (X’Pert PRO—PANAlytical, Eindhoven, The Netherland) CuKα, Ni-filtered radiation (λ = 1.54184 Å) are given in Figure 1a. Based on the chemical compositions in Table 1, the SS can be classified as calsialic for the white and ferricalsialic for the black slag specimens. The particle size distributions which were measured by a laser particle size instrument (MASTER SIZER 2000, Malvern, UK) arepresented in Figure 1b. The morphology of the grains as observed by ESEM is presented in Figure 1c. It is evident that the black slag is finer than the white sample in the range of small particles, up to 32 µm (Figure 1b). Between 32 and 283 µm the B specimen is finer and when the particle size was greater than 283 µm the black slag is coarser. The white slag with low iron content and highest CaO presents particles size similar to that of ordinary Portland cement [2] while the particle size distribution of the black sample is typical of steel slag. The iron oxide and RO phases constitute the essential of the coarse particles.

**Figure 2:**
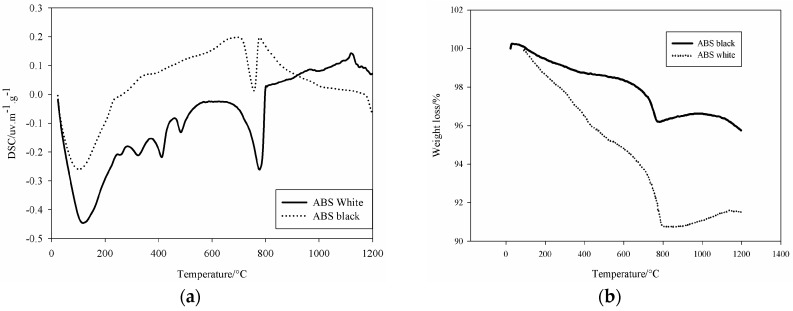
Thermal characterization of the starting two steel slags used for this study: (**a**) DSC curve and (**b**) TGA analyses.

The specific surface area as determined using nitrogen sorption method, BET (Brunauer-Emmet-Teller, method by nitrogen absorption on a GEMINI 2360, Micromeritics Instrument Corp., Norcross, GA, USA) gave 3.39 m^2^/g for the white ABS slag and 2.06 m^2^/g for the black ones. Under thermal analysis (Figure 2), both slags presented endothermic peak around 106 °C related to the physico-absorbed water present within the samples. White slag showed peaks of decomposition at 256, 324, 413, and 487 °C. Peaks consecutive to transformation of hydrated for of Fe_2_O_3_. xH_2_O, y-FeOH(OH) and Mg(OH)_2_ [4]. These compounds formed during the water granulation processes through the pouring into larger quantity of water to avoid excessive foaming. The peak situated at 760 °C corresponds to the decomposition of the Ca based phases of the slag. The peak at 1119 °C is related to the crystallization of aluminosilicate type mullite. The weight decrease for both steel slags (Figure 2b) is indicative for the percentage of volatile materials into the samples (Ca(OH)_2_, H_2_O, CO_2_, *etc.*).

The metakaolin (MK) was obtained from a standard kaolin used for glaze formulation in the ceramic industry after calcinations at 700 °C for 4 hand ground finely down to 30 µm (D90). The oxide molar composition of the metakaolin, determined by XRF, gave a value of Si/Al = 1.3 in terms of the mass ratio [13].

### 2.2. Mix-Design and Preparation of Inorganic Polymer Mortars

#### 2.2.1. Mix-Design

The white and black ABS slags presented 40.31 wt % and 36.52 wt % of CaO but the SiO_2_ contents of both materials were 11.33 wt % and 12.78 wt %, respectively. These contents result in Ca/Si molar ratios of 5.45 for the WSS and 4.38 for BSS. The main oxide content, *i.e.*, SiO_2_ + Al_2_O3 + Fe_2_O_3_, are less than 40 wt % for the white ABS and <50 wt % for the black sample, far from the ~80 wt % proposed by Davidovidts [14] as requirement for the formulation of polysialates.

We proposed to adjust the bulk chemical composition with addition of metakaolin, MK, in a mix with semi-crystalline particles of silica sand. The white and black ABS slags, SS, were used with metakaolin in the following proportions: 1 SS:1 MK(B60 and W60), 2 SS:1 MK (B90 and W90) and 3 SS:1 MK (B120 and W120). The obtained solid precursors were finely ground down to 60 µm and used to form inorganic polymer mortars with sand representing two times the mass of slag-metakaolin, SS-MK, powder. The sand was also ground to reduce the particles size below 400 µm for research sake, but to improve the economy of the process the natural sand can be replaced with fine gravel.

#### 2.2.2. Sample Preparation

NaOH (laboratory grade of pellets from Sigma Aldrich, Milan, Italy) was dissolved in deionized water to reach a concentrated 8M solution. This solution, after having been stored for a minimum of 48 h, was mixed with sodium silicate (SiO_2_/Na_2_O, molar ratio = 2.98; industrial grade from Ingessil, Verona, Italy) in volume proportion of 2:3. The final mix was used to prepare the inorganic polymer pastes with proportions reported in Table 2. The variation in the liquid: solid weight ratio (liquid = soda plus Na-silicate solution; solid = SS plus MK plus sand) as indicated into the Table 2 as well as the NaOH:Na_2_SiO_3_ volume ratio were chosen considering the behavior of the steel slag into the alkaline solution and the objective to draw pastes with similar final viscosity. The difference between the series B and W was dependant on the difference between the potential reactive fraction of each steel slag. The pastes for the six formulations were mixed in a bench mixer for approximately 10 min and poured in 1 × 1 × 14 cm^3^ polyurethane molds. Soon after pouring, the specimens were isolated from air into closed plastic films for the first 72 h. The curing continued in ambient conditions, room temperature around 21 ± 2 °C and 54% ± 1% of humidity (usual indoor environment) for up to 365 days. This curing procedure was considered optimal for the samples prepared in the laboratory with no defects. The mechanical characterization was performed on the specimens cured for 28 and 365 days.

### 2.3. Characterization of the Cured Steel Slag-BasedAlkali-Activated Mortars

The mineralogical analysis of the alkali-activated steel slag, AAS, mortars were carried out with an X-ray powder diffractometer, XRD (see above) from 5° to 70°, 2theta steps and integrated at the rate of 2 s per step.

Fourier transformed infrared spectroscopy, FT-IR, (Avatar 330 FTIR, Thermo Nicolet, Waltham, MA, USA) was performed on each sample analyzing fine powders of ground specimens (ф < 80 µm) collected from samples used for the mechanical test after curing for 28 days. A minimum of 32 scans between 4000 and 500 cm^−1^ were averaged for each spectrum at intervals of 1 cm^−1^.

An Autopore IV 9500, Micromeritics, 33000 psi (228 MPa) Mercury Intrusion Porosimeter (MIP, Manchester, UK) covering the pore diameter range from approximately 360 to 0.005 μm having two low-pressure ports and one high-pressure chamber was used for the pores analysis. Pieces were prepared from the bulk of each sample with specimens of ~1 cm^3^ of volume for the MIP. Apart from the pore volume and size, the equipment software also automatically evaluated the tortuosity as the ratio of the length of the path described by the pore space to the length of the shortest path across a porous mass.

Specimens (10 for each composition to reduce the error within 20%) with nominal size of 10 ± 0.10 mm in width, 10 ± 0.10 mm thick and a length of 140 ± 0.10 mm were used for the mechanical tests by using a three-point bending configuration with span equal to 40 mm according to ASTM C1161-02c standard. The specimens were loaded by a universal testing machine, type MTS 810, (MTS Systems Corporation, Eden Prairie, MN, USA) with cross-head speed of 5 mm/min.

The microstructure of the inorganic polymer cement specimens was studied using an Environmental Scanning Electron Microscope (ESEM, Model Quanta 200, FEI, Hillsboro, OR, USA) at low vacuum. Both fractured pieces from mechanical testing and etched polished specimens were used. The specimens were preliminarily coated with 10 nm thick gold layer. The ESEM was equipped with an Oxford Instruments energy dispersive spectrometer (EDS) for the microanalysis thus facilitating the investigation of phase distribution in the matrix.

To evaluate the capability to help in the control of indoor humidity in buildings, the adsorption/desorption behavior of the AAS mortars was evaluated on two specimens aged 28 days per each formulation dried at room temperature for 24 h and then soaked in deionizer water for 24 h accordingly to previous studies [15]. Specimens of prismatic shape as those used for mechanical characterization were weighted in their saturated condition and the time of the first measurement was considered as *t*_0_. After each hour—for the first 10 h—the measurement was repeated, then weight measurement of the sample continued every 24 h for 1000 h. This measurement was repeated four times (four cycles, also indicated as water cycles) in the laboratory conditions (T = 21 ± 2 °C, R.H. (Room Humidity) 54% ± 1%). Finally, water absorption was determined as ratio of the water saturated specimen with respect to the dry one; variation of the water release after saturation is expressed as rate of weight change and the humidity content coincides with the water retained by the sample after the 1000 h test time. Additionally, the shrinkage and expansion were monitored, however, changes in dimensions were not identified.

## 3. Results

### 3.1. Phases Development

**Figure 3:**
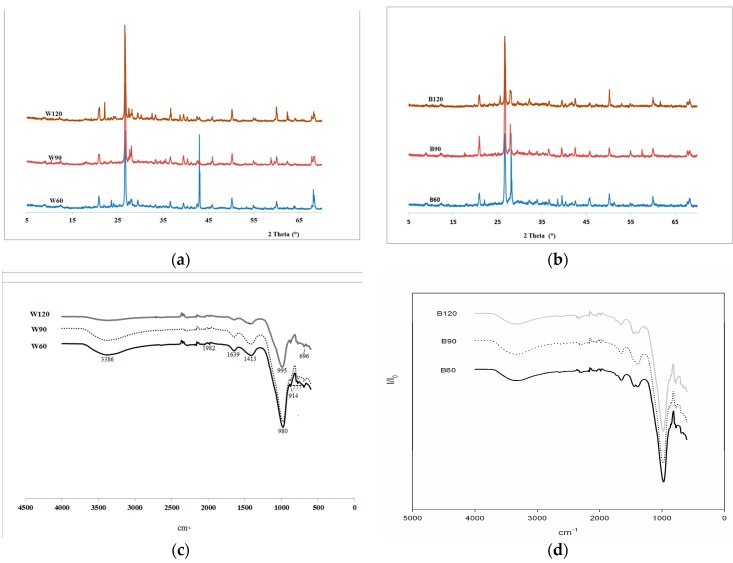
XRD and FT-IR characterization of the white and black slag-based AAS mortars: (**a**) XRD of white; and (**b**) black ABS; (**c**) FT-IR of white; and (**d**) black ABS.

The XRD patterns of both white and black slag-based AAS mortars exhibited amorphous structure and higher peak intensities of crystalline phases (Figure 3a,b). The halo that generally characterizes the fully reacted and amorphous structure of alkali-activated materials is visible around 2θ = 27° for all formulations. These broad diffuse halos in XRD patterns correspond to three-dimensional networks for low angle values (2θ < 30°) [5,16]. In our study, these halos were centered at 28.42°, 28.16° and 27.64° respectively for 1:1, 1:2 and 1:3 MK:WSS; and at 27.33°, 27.30° and 27.31° for MK:BSS. Typical metakaolin based inorganic polymer cement generally shows 2θ < 27° [17]. Thus it can be deduced that the introduction of the steel slag, with low aluminosilicate content, reduced the degree of typical N–A–S–H gel formation and polymerization of aluminosilicates (geopolymerization).The shift toward higher 2θ values by introducing steel slag is also confirmed by Bignozzi *et al.* [13] for geopolymers with ladle slag (white slag).

The formation of only amorphous phases peculiar of MK geopolymers is no longer ensured after the introduction of steel slags. In fact, the as-received steel slag are partially composed of crystalline phases, such as C_3_S, C_2_S, C_4_AF, and C_12_A_7_ as well as RO (R = Ca, Fe, Mn) solid solution [18], which contributed to modify the level of disorder and crystallinity into matrix. Their participation into the geopolymerization process is limited to the surface reactivity that contribute to the densification of the mortars, nevertheless no quantitative calculation of the crystallinity/amorphicity ratio was done in this study.

From the FT-IR spectra (Figure 3c,d), it is observed that all samples show two bands related to O–H stretching: (i) at 3347 cm^−1^ for B series and 3386 cm^−1^ for W series; and (ii) ~1646 cm^−1^ for W series and 1654 cm^−1^ for B series. The increase in steel slag content decrease the intensity of both peaks related to the O–H stretching. This is related to the reduction of typical N–A–S–H phases from metakaolin-based geopolymers that decrease with the increase of the steel slag. The behavior of the bands can be correlated with the evolution of the peaks around 1400–1450 cm^−1^, generally ascribed to three-fold-coordinated aluminum that decreases in intensity with the increase of the steel slag content (Figure 3c,d). B60 and W60, which presented the higher intensity for the OH-stretching peaks, have the principal peaks of alkali-activated aluminosilicates (Si–O–Si(Al)) at ~980 cm^−1^ and 982 cm^−1^ respectively [19].

By increasing the slag content, the peaks of B series moves to 994 cm^−1^ for B120 and 995 cm^−1^ for W120. The shift of the principal band towards higher wavenumber with the increase of the steel slag content indicates that with the introduction of the slag, very limited content of aluminum available and the possibility of their incorporation into the geopolymer network decrease [19,20,21,22,23]. The bands with small intensities between 690 and 850 cm^−1^ are attributed to geopolymer products. They are related to the symmetric stretching vibration of Si–O–Si (or Si–O–Al) bridges as well as the cyclosilicates vibrations [19,20]. They are also affected by the variation of the slag content. The band between 880 and ~900 cm^−1^, presented as a small shoulder on the main Si–O–T band, is assigned to the stretching vibration mode of Al–O bonds in condensed AlO_4_^−^ groups.

### 3.2. Porosity and Pore Size Distribution

#### 3.2.1. Pore Permeable to Mercury in MIP Tests

**Figure 4:**
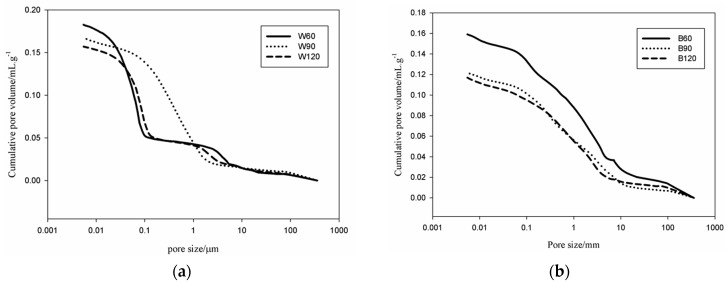
Cumulative pore volume of the slag-based AAS mortars: (**a**) white; and (**b**) black ABS.

The variations of the cumulative pore volume of the white and black slag-based AAS mortars are summarized in Figure 4. B60 presents 0.160 mL/g of cumulative pore volume. This value decreases to 0.122 mL/g and 0.118 mL/g, respectively for B90 and B120. A similar trend is observed for the W series: the cumulative pore volume decreases from 0.180 mL/g for W60 to 0.170 and 0.160 mL/g for W90 and W120, respectively. In general, the pores volume for the W series is more important than for B series. However, the pores volume in both inorganic polymer mortars is lower than in typical metakaolin based inorganic polymer cement (~0.300 mL/g) [21,22]. These values are pretty reliable as far as our experience is related since we usually operate after careful calibration of the equipment [17].

Focusing on the cumulative pores volume curves (Figure 4), the progressive decrease in the pore (larger capillary pores with size >1 µm) volume noted has important values for B120 with respect to B90. Similarly, the larger capillary pores in W120 are significantly bigger than in W90. The understanding of the relative decrease in cumulative pore volume with the increase of the slag content needs two important factors to be considered: (i) both slags contain a fraction of aggregates (27 wt % for the black slag and 64 wt % for the white one) which in this study are presented as fine aggregates [17]; and (ii) the role of metallic iron in the dissolution and polymerization reactions [23,24]. As described above, the finer particles of the steel slags are essentially composed of RO_ss_ (R = Ca, Fe, Mn, and Mg solid solution) of semi-crystalline nature. These fines undergo to incongruent dissolution, *i.e.*, they react essentially on the surface, during the dissolution, thus participating in the chemical-physical reactions typical of geopolymeric consolidation, reducing the volume of air voids and bubbles as generally observed in metakaolin-based inorganic polymer cement [15,19]. The role of fines in the reduction of pores volume has already been demonstrated by Kamseu *et al.* [15]. The fines into the context of inorganic reactions are known to accelerate the hydration acting as nucleation sites for binding phases (C–S–H, C–(N)–A–S–H, N–A–S–H, *etc.*) [25].

**Figure 5:**
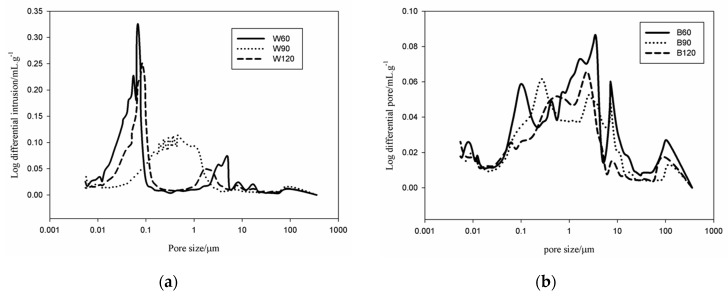
Pores size distribution of the slag-based AAS mortars: (**a**) white; and (**b**) black ABS.

The small particles of metals still present in the slags are corroded by the alkaline media enhancing the heat of reaction and thus the kinetic of the reactions. These metallic particles are also responsible for the increase of the fraction of the larger capillary pores due to hydrogen evolution similarly to what shown for the case of aluminum metal in concentrated alkaline solutions [26]. The major fraction of pores of the W series is concentrated between 0.01 and 0.1 µm (Figure 5). Such pores correspond to those generally described for the fly ash and metakaolin based inorganic polymer cements [17,21]. The coarser capillary pores with size between 1 and 10 µm that are not generally observed in the IPC, are ascribed to pores developed from the corrosion reactions of the metallic iron contained in the slag. This class of pores is more important for the B specimens than for W ones in agreement with the iron content accounting for 12.3 wt % and 31.9 wt % for W and B, respectively. In the W series the dominance of the gel pores (size between 0.01 and 0.1 µm) make the cumulative pore curves present the typical characteristics of the IPC mortars: apart from a small increase in the region of the capillary pores due to iron residues (1–10 µm), a significant increase is observed at 0.1 µm. The fraction of the pores between 1–10 µm in W series increased from 16.7 vol % for W60 to 17.6 vol % and 18.8 vol % for W90 and W120, respectively. From the behavior of the cumulative pores volume and the particles size distribution (Figure 4 and Figure 5) the two classes of pores into W series are isolated, hence the interconnection degree is very limited. In the B series the cumulative pore volume curves evidence the effect of the corrosion of metallic iron on the fraction of the pores >0.1 µm. Only ~16 vol % of pores have size <0.1 µm in B90 and B120 specimens, with the percentage raising to 18.8 vol % for B60. Pores with size >0.1 µm dominate the total porosity of the specimens of the B series. This is completely different in series W where most of the pores have size <0.1 µm. Consequently, the structure of the B series materials corresponds to a matrix with interconnected pores as confirmed in the Figure 4 and Figure 5. The increase of the fraction of larger capillary pores with the steel slag content in both B and W series confirms the role of the metallic iron during the alkali-activation of the steel slag mortars. The difference between the two samples shows that the Fe particles action on porosity can be tuned using the mix-design.

#### 3.2.2. Pores Permeable to the Moisture and Liquid Water

**Figure 6:**
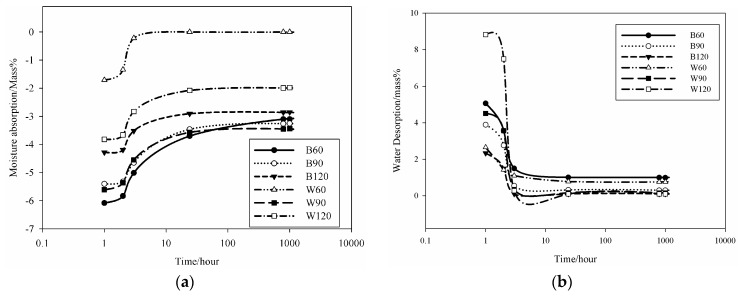
Moisture absorption-desorption performance of the slag-based AAS mortars: (**a**) moisture absorption performance at ambient temperature after 24 h in oven at 100 °C; and (**b**) moisture desorption performance at ambient temperature after complete saturation in water.

After 365 days curing at room temperature, the white and black slag-based AAS mortars release 2.5 wt %, 5.9 wt % and 4.1 wt % moisture for W60, W90 and W120, respectively, when treated at 100 °C for 24 h. The moisture released in similar conditions for B60, B90 and B120 were 6.6 wt %, 5.7 wt % and 4.4 wt % respectively. It is clear that moisture content increases with the amount of white slag while the behavior is opposite with black slag. W60 specimen with the lower moisture release (2.5 wt %) possesses the higher specific cumulative pore volume (0.18 mL/g). B60 specimen with the higher moisture release (6.6 wt %) presents the highest volume of larger capillary pores with size >1 µm. It is observed that the specimens of the series B reabsorbed the moisture with a higher rate compared to the specimens of the W series (Figure 6). These observations are correlated to the pore network structure of the two AAS mortars: B series samples present a larger fraction of pores with size >0.1 µm (>70 vol %) while W series specimens possess only ~16 vol % pores with size >0.1 µm and more than 80 vol % pores with size <0.1 µm. The large fraction of pores with size >0.1 µm explains the higher degree of pore connectivity in series B with respect to series W. Specifically, the fraction of pores with size between 1 and 10 µm that are supposed to be developed from the Fe particles corrosion during the alkali-activated reactions (Figure 4 and Figure 5) are more important in the series B. The important volume of these pores can be considered as the origin of pores coalescence, hence significantly reducing the gel pores. B60, B90 and B120 specimens cured at room temperature for 365 days at ambient relative humidity (R.H. 54% ± 1%) absorb 5.9 wt %, 4.5 wt % and 3.3 wt % water, respectively,. This decrease in water absorption with the amount of steel slag for B series can be correlated with the decrease of the cumulative pore volume (Figure 4).

Water absorption of the W series is 3.5%, 5.5%, 9.7% for W60, W90 and W120, respectively. It is observed that the presence of steel slag in W samples increases the ability to absorb water, as it can be appreciated from the variation of the moisture content (Figure 6). These results demonstrate that the permeability of the steel slag based mortars can be interpreted for B samples with the cumulative pore volume variation: the reduction of the cumulative pore volume implies a reduction of the water absorption.

The reduction of the water absorption is in agreement with the reduction of the volume of the larger capillary pores with size >0.1 µm. In the W series, although the cumulative pore volume reduces with the white steel slag, water absorption increases. Here, a better correlation is made between water absorption and the volume of the larger capillary pores. This volume increases from 16.7% for W60 to 17.6% and 18.8%, for W90 and W120, respectively. This means that the permeability in W series is mostly affected by the development of larger capillary pores from the corrosion in the pore solution of the iron metal particles. Finally, it can be mentioned that the presence of the steel slag reduces the capacity of the metakaolin based geopolymer to accumulate moisture. The specimens with SS:MK = 1:1 (B60 e W60) remain after 1000 h with ~1 wt % moisture at room temperature while specimens with SS:MK = 2:1 and 3:1 (B90, B120, W90 and W120) reduce the moisture content down to ~0.25 wt %.

The collected results make this class of slag (EAF white and black steel slag) as promising additive materials for the moisture control capacity of geopolymeric mortars. It seems that the mechanism of the moisture control capacity enhancement is focused essentially to the formation of the particular class of pores (1–10 µm) in the geopolymeric matrix.

### 3.3. Density and Flexural Strength

**Figure 7:**
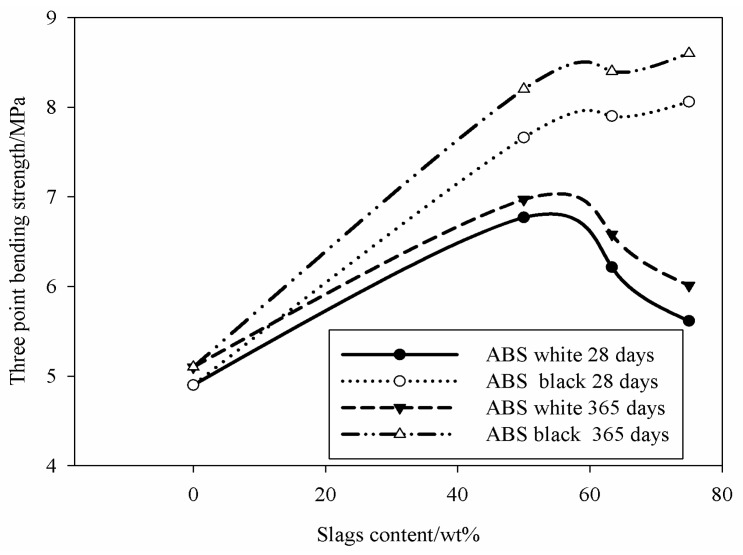
Three point bending strength of slag-based AAS mortars with white and black ABS after 28 and 365 days.

The physical and mechanical properties of the white and black slag-based AAS mortars are summarized in Table 3. In general, the bulk density of standard metakaolin based IPC is ~1.50 g/cm^3^ [27,28]. The presence of fine aggregates (64.1 wt % for W and 27.0 wt % for B) is the primary reason of the density increase. The presence of sand also plays a significant role together with the presence of iron (12.3 wt % for W and 31.9 wt % for B). The relative increase in density corresponds to higher flexural strength, raising from ~4 MPa [17,21] to 6.8 MPa for W60 specimen. White ABS contains higher aggregates and this favors the reactivity reduction and polycondensation, thus reducing the flexural strength (Figure 7). In addition, increasing the curing time up to 365 days does not change this tendency. ForB series the high reactivity of the black slag can explain the continuous increase of the polycondensation, thus increasing the flexural strength with the steel slag amount. The difference in the strength development between the white and black slag can be justified by different aggregates fractions (27 wt % for B sample and 64 wt % for W). Actually, since the two SS presented these two different sand fractions, we adopted different paste/sand ratios in order to optimize the final consolidated product in terms of chemical stability and strength. The optimum ratio corresponds to the best ITZ features.

Commenting the relationship between porosity and flexural strength, we can say that according to our experience on geopolymers and ceramics, we noticed that in general when the total porosity is determined with the mercury intrusion porosimeter, the values obtained are generally higher when compared to others methods. This is particularly true for geopolymers, due to the fact that the nanoporosity of these materials are takeninto account. At the same time we also noticed from literature data that this nanoporosity does not affect the materials during the mechanical solicitation.

Concerning the chemical reactivity and chemical bonding in relation to mechanical strength, we noticed that although the incongruent dissolution of these aggregates in the pore solution can be pointed out as well as their participation in the surface physical-chemical reactions, their high proportion limits the strength when amount of binder is not enough to embed the aggregates. On the other hand, both white and black steel slags show similar silica (SiO_2_) content. However, the white specimenscontain15 wt % Al_2_O_3_, three times larger than in the black ones (5 wt %) (Table 1). The high temperatures at which slags are produced (>1600 °C), allow the formation of more stable aluminosilicates as glass-ceramics in the white specimens. These phases have limited dissolution in alkaline media, meaning relatively low soluble silica and alumina and low gel formed for the polycondensation optimization. The relative increase in strength in both B and W series with curing time from 28 to 365 days is related to the additional pozzolanic reactions that take place with very low rate. The completely dissolved metakaolin and steel slag finer particles contribute to the formation of oligomers, that convert to polymerized small clusters of aluminosilicates or calcium silicates/calcium-aluminosilicate hydrated gels (5–10 nm) [17,29]. The effectiveness of these gels to enhance strength depends on the gels/aggregates ratio, their interfaces and chemical equilibrium. The aggregates dissolution level and the strain generated by the surrounding matrix play a significant role in determining the final strength since it affects cross-linking among phases. In the metakaolin based IPC, the reactive phases are generally N–A–S–H gels leading to a flexural strength near 4 MPa [17]. Increasing the available soluble silica (in the range of Si/Al = 2–2.5 molar ratio), the mechanical strength increases as a result of the pores volume and size reduction [17,29]. For both steel slags in W series, an improvement of the flexural strength was observed as the result of the cumulative pore volume reduction although the presence of a new class of pores between 1 and 10 µm is formed, (not significant) (Figure 5). The larger amount of pores with size >0.1 µm in B series limits the strength increase. The strengthening mechanism in this case includes a reduction of the cumulative pore volume. A possible improvement of the mechanical performance could have been reached extending the first step of curing of 72 h. The laboratory environment might have brought to excessive desaturation of samples in the very early age.

### 3.4. Microstructure

**Figure 8:**
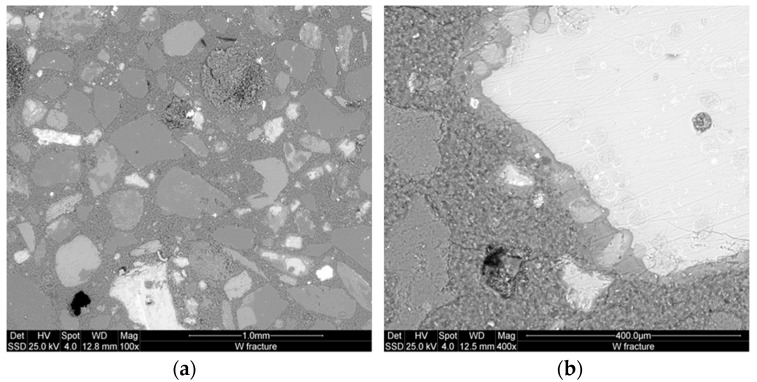
ESEM microstructure of white slag-based AAS mortars showing: (**a**) overall microstructure with quartz and iron-rich aggregates (solid solution) embedded into the matrix; (**b**) interface zone demonstrating good adhesion bonds between the various aggregates and the matrix.

**Figure 9:**
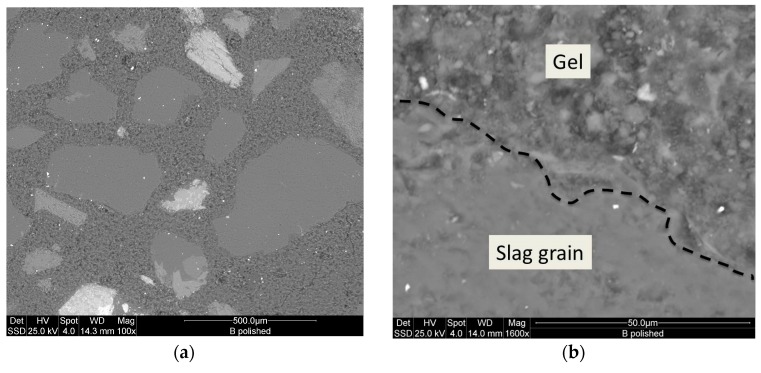
ESEM microstructure of black slag-based AAS mortars showing: (**a**) overall microstructure with quartz and iron-rich aggregates (solid-solution) embedded into the matrix; (**b**) interface zone demonstrating good adhesion bonds between aggregates.

**Figure 10:**
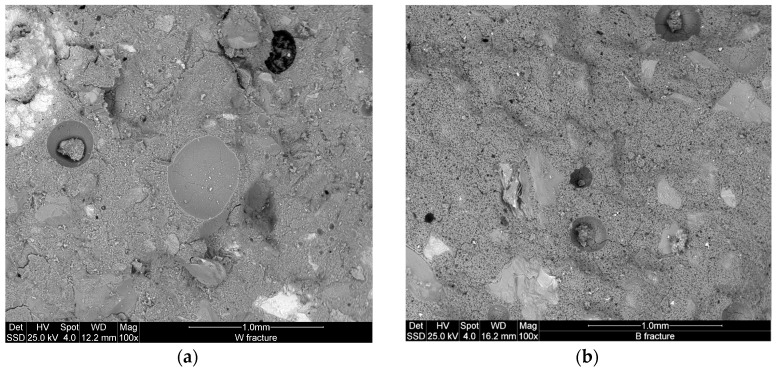
ESEMmicrostructure of fracture surface at low enlargements of the specimens with: (**a**) white ABS; (**b**) black ABS.

**Figure 11:**
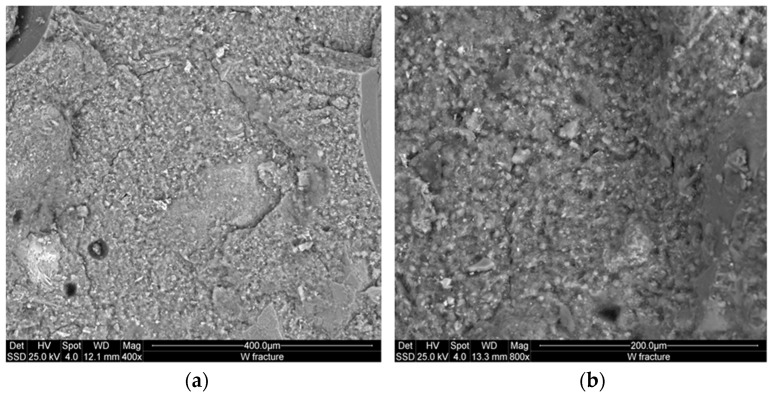
ESEM microstructure of fracture surface at high enlargements of the specimens with white ABS: (**a**) 400×; (**b**) 800× showing the relative poor impact of the low concentration of the iron metal during the process.

**Figure 12:**
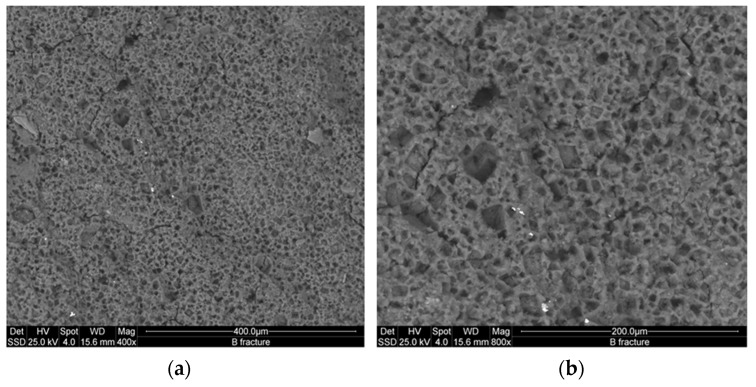
ESEM microstructure of fracture surface showing the homogeneous distribution of the pores with size between 0.1 and 10 µm forming from the corrosion of the iron metal during the process of black ABS mortars: (**a**) 400×; (**b**) 800× SS-IPC.

Figure 8 and Figure 9 show the microstructure of the inorganic polymer mortars with ABS white and ABS black, respectively. The micrographs, taken on polished samples, point out larger quartz grains (dense and compact) and the aggregates form the steel slag (highly porous). The matrix is compact and homogeneous with well-embedded aggregates. At limited magnification, no crack can be observed. The fracture surface of the specimens with white (Figure 10a) and black ABS (Figure 10b) show that the aggregates are well covered by the geopolymer matrix composed of calcium-aluminosilicate gels. In this case, aggregates are not clearly visible, same as in the polished samples: this demonstrates the good capacity of the steel slag based IPC to embed aggregates with significant adhesion bond strength. At higher magnification (Figure 11 and Figure 12), the microporous network structure in B series can observed. The matrix is dominated by homogeneously dispersed pores with sizes in the range of 1 to 10 µm. This is the origin of the difference in microstructure between B and W series. Pores between 1 and 10 µm can also be observed in W specimens; however, the concentration is too low to be relevant for the mechanical performance of the matrix.

As already mentioned, the importance of the specific pores range (1–10 µm) is directly related to the iron content, which is 12.3 wt % for the ABS white and 31.9 wt % for the ABS black. Additionally, the residues of metallic iron formed in the slag are corroded by the NaOH solution during the dissolution phase of geopolymerization. The evolved gas is responsible for the formation of pores with size between 1 and 10 µm for W series and 0.1 and 10 µm for B series.

The homogeneous dispersion of pores from the exothermic reactions of iron corrosion is due to the efficient mixing operation of steel slag, metakaolin and sand particles for the preparation of the AAS paste. Jointly with the proper mixing operation, the presence of iron particles in a homogeneous mortar system resulted well distributed in the way to produce the corrosion reaction at isolated sites avoiding extensive pores coalescence. The limited fraction of the metallic form of the iron justified the behavior observed for both B and W series, althoughthe micrographs of the B series although this class of interpores demonstrated a high compactness into the interpores partitions (Figure 11 and Figure 12). The W series maintained the global compactness of the conventional IPC mortars as a result of dissolution of the major fraction of the slag to highly reactive oligomer species. The oligomers participate in the formation of new phases: N–A–S–H, C–(A)–S–H which form, with the residues of non-reacted phases, a compact structure due to the adhesion bonds capacity of these particles as well as that of the river sand. The steel slag dissolution allows the formation of Fe^3+^ ions that also participate to the formation of geopolymeric phases with their insertion into the network structure of N–A–S–H or C–(A)–S–H resulting into ferrosialtes [14]. The similarity of Fe^3+^ with Al^3+^ ion, explains why Fe^3+^ acts as network former that can occupied a tetrahedric site. Both the energy of the dissolution of the metallic iron and the potential insertion of its cations into the geopolymer network can explain the strength enhancement observed for the AAS mortars, mainlyin the BSS formulations that shows higher strength, although the considerable fraction of capillary pores between 1 and 10 µm (Figure 7). The RO crystalline particles in the steel slag refer to crystalline Fe^2+^ bearing mineral phases as ferronforsterite, augite, and others pyroxene type.

According to Lemougna *et al.* [23], a portion of these minerals, as augite, react with the alkaline solution to form new ferric sites which may be located in amorphous gel-like phase. This situation may explain why the steel slag to metakaolin mass ratio did not appear as the significant parameter governing the geopolymerization and strength behavior of the steel slag mortars. It is suggested that in the formulations range adopted in this study, the increase of slag content reduces the alumina oligomers formed during the dissolution process due to the reduction of metakaolinthat provides the alumina oligomers inthe mortars. Thus a fully reacted and cured matrix of the steel slag based inorganic polymer mortars can then be described as a continuous and homogeneous solid solution (gel-like structure) comparable to that based on metakaolin or fly ash, with the particularity of the pore network that can be controlled with mix-design and compensated with the strengthening effects of the iron into the adhesive bonds. The presence of metakaolin and semi-crystalline silica sand has the effects to increase the homogeneity and the compactness of the microstructure avoiding micro cracks and unreacted alkali [17,21].

## 4. Discussion and Conclusions

Astutingsih and Liu [30] indicated that in alkali-activated slag, alkali ions (K^+^, Na^+^, *etc.*) are located at a non-bridging oxygen site [31,32,33]. The use of amorphous alumina and silica from metakaolin and semi-crystalline silica sand generates structural matrices with high flexural strength as the result of the cracks stabilization, reduction of the cumulative pore volume and improvement of the microstructure. The strengthening mechanism is focused on the existence of both N–A–S–H and C–N–(A)–H suitable adhesive bonds to embed the various aggregates in the matrix. The metallic particles content of the various mix acts at first through its corrosion in alkaline environment to enhance the reactions heat thus densifying the inter-pores partitions although the porosity between 1 and 10 µm results from the iron activities. Fe^3+^ and Fe^2+^ from the corrosive reactions and the dissolution of some RO minerals react with the alkaline solution to form new ferric sites which may be located at amorphous gel-like phase [23].

Kotama *et al.* [34] correlated the moisture control capacity with pore size distribution makethe difference between the gel pores and the capillary pores. They designed ideal construction materials with pores distributed between those with size <0.1 µm, considered as pores responsible for the absorption, and capillary pores (0.1 µm < ф < 10 µm) responsible for desorption. The detailed analysis of the pores in the AAS mortars show that the BSS series present 15.6 vol % for B60 and 17 vol % for B90 and B120 of pores with size 0.1 µm. For WSS series, apart from W90, the specimens present pores having 60% with size <0.1 µm. The analysis of moisture absorption–desorption performance indicates the potential absorption up to ~6% moisture in the context of maximum humidity. In general, increasing the gel pores content (ф < 0.1 µm) will increase moisture absorption. Pores with size between 0.1 and 10 µm contribute to enhance the moisture desorption capacity. In fact, the moisture absorbed was found to be desorbed almost totally within ~8 h. Less than 2.5 vol % moisture remains in B60 and W60. The moisture percentage decreases below 0.5 vol % for B90, B120, W90 and W120. It can be concluded that the presence of the steel slag improves the moisture control capacity leading to the production of pore network that enable regulation of absorption and desorption within a short range of time (Figure 6). The above described pore network allows: (i) no accumulation of moisture within the bulk structure of the mortars and by the way high exchange capacity; and (ii) by using the geopolymer process technology, the surface thickness described by Kotama *et al.* [34] can be achieved with the molding design in relation to the viscosity of the alkali-activated pastes making the surface thick enough to avoid the ingress of aggressive agents while improving the exchange rate between the environment and the bulk.

The above-described formulations based on the steel slag with particular class of capillary porosity have shown that alkali activation accompanied by an appropriate mix design is a procedure capable of reusing a high amount of end-of-waste materials or byproducts as steel slag. Apart from the low embodied energy, since the activation of slag and its consolidation required very low amount of energy with respect to the energy required for conventional fired bricks and cement clinker, these AAS mortars also showed an interesting adsorption/desorption behavior that suggested their use as coating material to maintain the stability of indoor relative humidity.

All of these characteristics are in line with the challenges of the EU Framework Programme Horizon 2020 [35] for the eco-efficient construction and building materials research.

## Figures and Tables

**Table 1:** Chemical compositions (wt %) of black and white steel slag (EUROPEAN STANDARD EN 15309 by XRF).

Oxides	ABS Black	Oxides	ABS White
CaO	36.52	CaO	40.31
Fe_2_O_3_	31.87	Fe_2_O_3_	12.28
SiO_2_	12.78	SiO_2_	11.33
MnO	5.90	MnO	2.19
Al_2_O_3_	5.04	Al_2_O_3_	15.43
MgO	3.26	MgO	11.54
Cr_2_O_3_	2.15	Cr_2_O_3_	0.73
TiO_2_	0.41	TiO_2_	0.31
SO_3_	0.31	SO_3_	1.68
V_2_O_5_	0.28	Be...F	0.36
P_2_O_5_	0.50	Others	3.84
Others	0.98		

**Table 2:** Mix-design of steel slag (SS) inorganic polymer mortars with similar value in the cone-fluidity test.

Formulation	SS:MK ^1^	(Slag + MK):Sand	Solid:Liquid	NaOH:Na_2_SiO_3_
B60	1:1	1:2	1.3:0.4	2:3
B90	2:1	1:2	1.3:0.4	2:3
B120	3:1	1:2	1.3:0.4	2:3
W60	1:1	1:2	1:0.4	2:3
W90	2:1	1:2	1:0.4	2:3
W120	3:1	1:2	1:0.4	2:3

^1^: MKmetakaolin.

**Table 3:** Microstructural data as determined by MIP of all the formulations aged 28 daysand mechanical properties at 1 year aging.

Formulation	Bulk Density (g·cm^−3^)	Median Pore Diameter (µm)	Total Porosity/%	Tortuosity (a.u.)	Flexural Strength at 365 Days (MPa)
B60	2.13	1.4	34.0	29.6	7.7
B90	2.22	0.8	29.0	39.8	6.9
B120	2.39	0.9	28.1	39.0	8.1
W60	1.74	0.1	31.9	38.1	6.8
W90	1.77	0.1	29.8	42.4	6.2
W120	1.82	0.1	28.6	49.8	5.6
